# Pre-treatment serum albumin predicts relapse in idiopathic inflammatory myopathies: a retrospective cohort study with cytokine profiling

**DOI:** 10.1093/rap/rkag021

**Published:** 2026-02-05

**Authors:** Haruna Matsuo, Toshimasa Shimizu, Tomohiro Koga, Noriho Sakamoto, Atsushi Kawakami

**Affiliations:** Division of Advanced Preventive Medical Sciences, Department of Immunology and Rheumatology, Nagasaki University Graduate School of Biomedical Sciences, Nagasaki, Japan; Division of Advanced Preventive Medical Sciences, Department of Immunology and Rheumatology, Nagasaki University Graduate School of Biomedical Sciences, Nagasaki, Japan; Division of Advanced Preventive Medical Sciences, Department of Immunology and Rheumatology, Nagasaki University Graduate School of Biomedical Sciences, Nagasaki, Japan; Department of Respiratory Medicine, Nagasaki University Graduate School of Biomedical Sciences, Nagasaki, Japan; Division of Advanced Preventive Medical Sciences, Department of Immunology and Rheumatology, Nagasaki University Graduate School of Biomedical Sciences, Nagasaki, Japan

**Keywords:** idiopathic inflammatory myopathies, relapse, albumin, cytokines, biomarkers

## Abstract

**Objective:**

To identify predictors of relapse in patients with idiopathic inflammatory myopathies (IIMs) and investigate the biological mechanisms underlying these associations using cytokine profiling.

**Methods:**

We conducted a retrospective study of 99 patients who achieved remission after initial treatment for IIMs or myositis-specific antibody-positive interstitial lung disease at Nagasaki University Hospital. Among 197 diagnosed patients, we included those who achieved clinical improvement and were followed up for ≥3 months, excluding early deaths and those with insufficient follow-up. Relapse was defined as disease worsening requiring treatment intensification. Risk factors were analyzed using the Cox regression analysis. Serum cytokine profiles were compared between patients with low and high pre-treatment albumin levels.

**Results:**

Forty-one patients (41%) relapsed. Multivariate analysis revealed that pretreatment albumin level remained the only independent predictor of overall relapse (HR, 0.60; 95% CI: 0.40–0.92, *P* = 0.019). For ILD relapse, both albumin (HR: 0.51, 95% CI: 0.30–0.88, *P* = 0.015) and female sex (HR: 0.70, 95% CI: 0.52–0.95, *P* = 0.022) remained independent predictors. In antibody-specific analyses, the V-neck sign strongly predicted ILD relapse in anti-ARS-positive patients (HR, 5.07; 95% CI: 1.64–15.75, *P* = 0.006). After false discovery rate correction, 14 cytokines remained significantly elevated in patients with low albumin levels, including IL-6 (7.5-fold, q < 0.001), IP-10 (5.3-fold, q < 0.001) and MCP-1 (2.5-fold, q < 0.001).

**Conclusion:**

Pretreatment albumin levels independently predict relapse in IIMs, and cytokine profiling demonstrates that low albumin levels reflect active systemic inflammation. These findings support the use of albumin as a practical biomarker for risk stratification in clinical practice.

Key messages

- Pretreatment serum albumin levels independently predict relapse in idiopathic inflammatory myopathies.
- Low albumin levels reflect systemic inflammation with elevated IL-6, IP-10 and chemokine levels.
- Cutaneous findings predict ILD relapse differently across the antibody subgroups.

## Introduction

Idiopathic inflammatory myopathies (IIMs) are a heterogeneous group of systemic autoimmune disorders primarily characterized by muscle inflammation and weakness, cutaneous manifestations and interstitial lung disease (ILD) [1, 2]. The major clinical subgroups include polymyositis (PM), dermatomyositis (DM), clinically amyopathic dermatomyositis (CADM) and anti-synthetase syndrome (ASS) [3]. The discovery of myositis-specific antibodies (MSAs) has further enhanced our understanding of these conditions and their distinct clinical phenotypes [4].

Despite advances in treatment strategies, including the use of glucocorticoids and various immunosuppressive agents, disease relapse remains a significant challenge in the management of IIMs [5]. Relapses can lead to cumulative organ damage, increased morbidity and reduced quality of life [6]. Studies have reported relapse rates ranging from 20% to 60% in patients with IIM; however, the factors contributing to these relapses are not well understood [7].

The presence of certain MSAs, particularly anti-aminoacyl tRNA synthetase (anti-ARS), anti-melanoma differentiation-associated gene 5 (anti-MDA5), anti-transcription intermediary factor 1-gamma (anti-TIF1-γ) and anti-signal recognition particle (anti-SRP) antibodies, is associated with distinct clinical features and disease courses [8, 9]. However, their predictive value for disease relapse has not yet been fully established. Furthermore, although various clinical and laboratory parameters have been suggested as potential predictors of disease outcomes [10, 11], there is currently no consensus on reliable markers for identifying patients at high risk of relapse. The ability to predict which patients are more likely to experience disease relapse would be invaluable for clinical decision-making, including the intensity and duration of immunosuppressive therapy and the frequency of monitoring. This knowledge could potentially help prevent relapses through more personalized treatment approaches and closer monitoring of high-risk patients.

Therefore, this study aimed to identify the clinical characteristics and risk factors associated with relapse in patients with IIMs by analyzing a cohort of patients from our institution and investigating the biological basis of these associations through comprehensive cytokine profiling. We particularly focused on the relationship between specific clinical features, laboratory parameters, MSAs, cytokine levels and the risk of different types of relapse (muscle/skin symptoms vs ILD).

## Methods

### Study design and patient population

This retrospective cohort study included patients diagnosed with IIMs or myositis-specific antibody-positive interstitial lung disease who visited the Department of Immunology and Rheumatology and Department of Respiratory Medicine at Nagasaki University Hospital between August 2008 and April 2024. For this study, which focused on relapse prediction during long-term follow-up, we included patients who met the following criteria: (1) received immunosuppressive treatment with initial response and clinical improvement (remission induction) and (2) were subsequently followed up at our outpatient clinic for at least 3 months. Immunosuppressive treatment with initial response and clinical improvement (remission induction) was defined as a sustained clinical response for at least 3 months after treatment initiation, characterized by improvement or stabilization of organ-specific manifestations, absence of treatment escalation during this period and objective improvement or stabilization of relevant laboratory and imaging findings. The study protocol was approved by the Institutional Review Board of Nagasaki University Hospital (approval number: 23082128), and the study was conducted in accordance with the Declaration of Helsinki. Written informed consent was obtained from all participants prior to enrolment.

### Diagnostic criteria and clinical assessment

IIM diagnoses were established using multiple, validated criteria sets. PM and DM were diagnosed based on the Bohan and Peter criteria [12]. CADM was diagnosed according to Sontheimer’s criteria [13]. The 2017 EULAR/ACR classification criteria [2] were applied to classify patients diagnosed after its publication. ASS was diagnosed in patients with antisynthetase antibodies in conjunction with one or more of the following features: myositis, ILD, arthritis, Raynaud’s phenomenon, mechanic’s hands and fever. Clinical features, laboratory data and treatment information were collected from the medical records. Myositis-specific antibodies were measured using commercially available ELISA kits (MESACUP™ Myositis ELISA series; Medical & Biological Laboratories Co., Ltd, Nagoya, Japan). The antibodies assessed included anti-ARS, anti-MDA5, anti-TIF1-γ, anti-Mi-2 and anti-SRP antibodies. ILD was diagnosed based on chest high-resolution computed tomography findings by a respiratory specialist.

### Definition of relapse

Disease relapse was defined as biochemical, clinical or imaging evidence of disease worsening that required intensification of immunosuppressive treatment, as determined by the attending physician. Muscle and skin relapses were identified based on biochemical (elevated muscle enzymes, including creatine kinase and aldolase) and/or clinical relapse (new or worsening muscle weakness or cutaneous manifestations). Interstitial lung disease relapse was determined by imaging relapse, defined as progressive interstitial lung disease on chest computed tomography (CT) scans, with or without accompanying respiratory symptoms.

### Data collection

At the time of diagnosis, we collected demographic information, including age and sex, along with clinical manifestations such as muscle weakness, skin findings (Gottron’s sign, heliotrope rash, mechanic’s hand and V-neck sign) and the presence of ILD. Laboratory parameters, including CK, CRP, ferritin, LDH, albumin and KL-6 levels, were recorded. Treatment details, including the initial prednisolone dose and the use of various immunosuppressants, were documented. Initial treatment regimens were classified into three categories: prednisolone monotherapy, combination therapy with prednisolone and other immunosuppressants, and triple therapy (high-dose glucocorticoids, calcineurin inhibitors and intravenous cyclophosphamide). We tracked the time to relapse and total duration of observation for each patient from the initiation of treatment.

### Serum cytokine measurement

Baseline serum samples were available for 71 patients in this study. Cytokine and chemokine concentrations were measured using the MILLIPLEX MAP Human Cytokine/Chemokine Magnetic Bead Panel (Millipore, Billerica, MA, USA). Of these, five cytokines with undetectable levels in more than half of the samples were excluded from the analysis. To explore the biological basis of the association between albumin and relapse risk, we compared cytokine profiles between patients with low albumin (<3.65 g/dl, below the median) and high albumin (≥3.65 g/dl) levels using Mann–Whitney *U*-tests. Given the exploratory nature of this analysis and the large number of comparisons, false discovery rate (FDR) correction was applied using the Benjamini–Hochberg method, with *q*-values <0.05 considered statistically significant.

### Statistical analysis

Baseline characteristics were summarized using descriptive statistics. Continuous variables are expressed as medians with interquartile ranges (IQR), and categorical variables are presented as numbers with percentages.

We conducted a risk factor analysis for relapse using Cox proportional hazards regression models. First, univariate Cox regression analysis was performed to identify factors associated with relapse, calculating the hazard ratios (HRs) with 95% confidence intervals (CIs). Subsequently, a multivariate Cox regression analysis was conducted to identify independent risk factors. The variables included in the multivariate models were selected based on clinical importance and statistical significance in the univariate analysis (*P* < 0.05), while considering the number of events to avoid overfitting. Given the strong biological and statistical correlations among acute-phase cytokines and inflammatory markers (e.g. IL-6 with CRP and albumin), multivariable cytokine modeling was not performed because it may introduce multicollinearity and over-adjustment, leading to unstable estimates and limited interpretability.

We analyzed relapse-free survival using the Kaplan–Meier method. Statistical significance was set at *P* < 0.05. All statistical analyses were performed using JMP version 18.0 (SAS Institute, Inc., Cary, NC, USA).

## Results

### Patient characteristics and relapse rates

**Figure 1:**
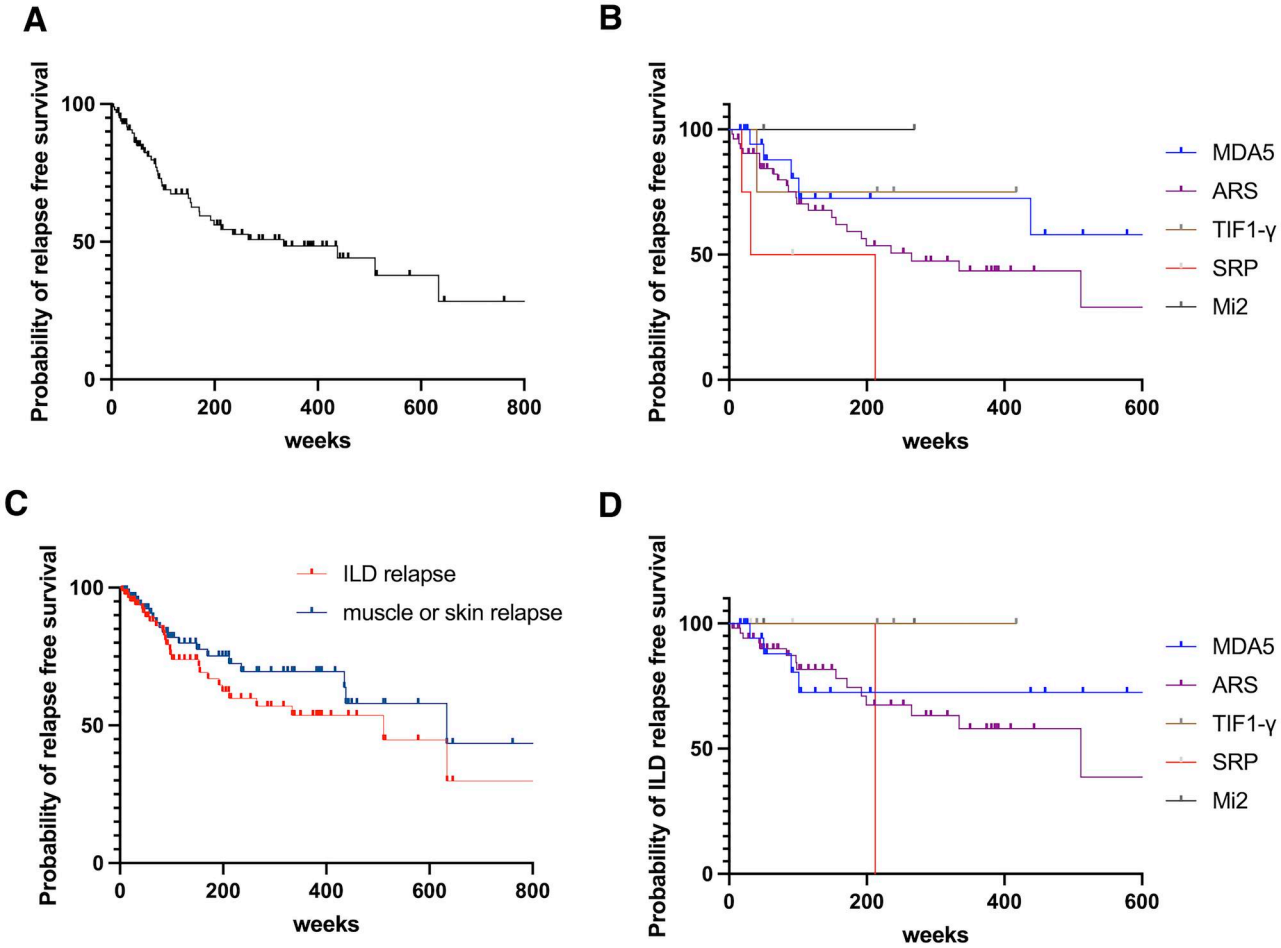
Kaplan–Meier curves for time to disease relapse in patients with idiopathic inflammatory myopathies. (**A**) Relapse-free survival in all patients (*n* = 99). (**B**) Relapse-free survival according to antibody status: anti-ARS, anti-MDA5, anti-TIF1-γ, anti-SRP and anti-Mi-2. (**C**) Relapse-free survival according to relapse type: ILD relapse versus muscle/skin relapse. (**D**) ILD relapse-free survival according to myositis-specific antibody profiles. The tick marks indicate the censored observations. ARS, aminoacyl-tRNA synthetase; ILD, interstitial lung disease; MDA5, melanoma differentiation-associated gene 5

During the observation period, 197 patients were diagnosed with IIMs or myositis-specific antibody-positive ILD. We excluded 98 patients for the following reasons: incomplete clinical data (*n* = 73), early death before achieving remission (*n* = 8) and insufficient follow-up duration (<3 months) due to transfer to other hospitals or loss to follow-up (*n* = 17). Forty-one of the 99 patients (41.4%) relapsed. The median time to relapse was 88 weeks (interquartile range [IQR]: 44–170 weeks). The overall relapse-free survival curve is shown in Fig. 1A. Relapse rates varied significantly according to autoantibody status. Patients with anti-ARS antibodies demonstrated a notably higher relapse rate of 54% (24/44 patients), whereas those with anti-MDA5 antibodies had a lower relapse rate of 21% (6/28). Patients with anti-TIF1-γ and anti-Mi-2 antibodies demonstrated relatively favorable relapse-free survival (1/4, 0/3, respectively), while patients with anti-SRP antibodies tended to experience early relapse (3/4). Among patients who experienced relapse, 20 (48.8%) had relapses involving muscle and skin symptoms, whereas 27 (65.9%) experienced ILD relapse. Notably, some patients experienced relapse in both categories. Relapse-free survival by antibody subgroup is shown in Fig. 1B, and organ-specific relapse-free survival curves in Fig. 1C. When ILD relapse-free survival was compared between anti-ARS- and anti-MDA5-positive patients, no significant difference was observed (log-rank *P* = 0.80; Fig. 1D).

The baseline characteristics of the patients stratified by relapse status are presented in Table 1. The median age was 61 years (IQR: 53–69 years), and 60% of the patients were female. The cohort included patients with polymyositis (7%), dermatomyositis (36%), clinically amyopathic dermatomyositis (30%) and antisynthetase syndrome (26%). Interstitial lung disease was present in 82% of the patients at baseline. The median observation period was 245 weeks (IQR: 86–403 weeks) for all patients, 302 weeks (IQR: 196–451 weeks) for the relapse group and 175 weeks (IQR: 66–385 weeks) for the non-relapse group.

**Table 1:** Baseline characteristics of patients with idiopathic inflammatory myopathies stratified by relapse status.

	All patients (n = 99)	With all relapse (n = 41)	Without relapse (n = 58)
Age (years)	61 (53–69)	61 (51–69)	61 (54–67)
Female (%)	59 (60)	24 (58.5)	35 (60.3)
PM (%)	7 (7)	4 (9.8)	2 (3.4)
DM (%)	36 (36)	14 (34)	22 (38)
CADM (%)	30 (30)	10 (24)	20 (34)
ASS (%)	26 (26)	13 (32)	13 (22)
Anti-ARS antibody (%)	53 (54)	24 (59)	29 (50)
Anti-MDA5 antibody (%)	21 (21)	6 (15)	15 (26)
Anti-Tif1γ antibody (%)	9 (9)	2 (5)	7 (12)
Anti-Mi2 antibody (%)	4 (4)	0 (0)	4 (7)
Anti-SRP antibody (%)	4 (4)	4 (10)	0 (0)
Muscle weakness (%)	52 (53)	24 (59)	30 (52)
Interstitial lung disease (%)	82 (82)	35 (85)	47 (81)
Malignancy (%)	24 (24)	11 (27)	13 (22)
Gottron’s sign (%)	63 (64)	23 (56)	40 (69)
Heliotrope rash (%)	18 (18)	8 (20)	10 (17)
Mechanic’s hand (%)	49 (49)	23 (56)	26 (45)
V neck sign (%)	24 (24)	10 (24)	14 (24)
CK (IU/l)	238 (88–836)	395 (154–839)	166 (75.5–748)
CRP (mg/dl)	0 (0.09–1.13)	0 (0.12–1.19)	0 (0.08–0.93)
Ferritin (ng/ml)	336 (145–539)	257 (116–439)	418 (161–570)
LDH (IU/l)	321 (227–389)	333 (249–410)	319 (210–387)
Alb (g/dl)	3.7 (3.2–4)	3.7 (3.2–3.9)	4 (3.2–4.1)
KL-6 (U/ml)	730 (447–1189)	727 (422.5–1212.5)	733 (495–1162)
Initial daily dose of PSL (mg/day)	40 (30–50)	40 (40–50)	40 (30–50)
Daily dose of PSL at relapse (mg/day)	7 (4–10)	7 (4–10)	–
**Initial treatment**			
Only PSL	23(23)	10(24)	13 (22)
Triple therapy	14(14)	4(10)	10 (17)
PSL+IS	62(63)	27(66)	35 (60)
**Immunosuppressant**			
IVCY (%)	15 (15)	4 (9.6)	11 (20)
CNI (%)	70 (71)	30 (73)	40 (73)
AZA (%)	4 (4)	2 (4.9)	2 (34)
MTX (%)	2 (2)	2 (5)	0 (0)
MMF (%)	1 (1)	1 (2)	0 (0)
RTX (%)	1 (1)	1 (2)	0 (0)
JAK-I (%)	3 (3)	0 (0)	3 (5)
IVIG (%)	7 (7)	7 (17)	3 (5)
Time to relapse (weeks)	–	88 (43.6–170)	–
Duration of observation (weeks)	245 (86–403)	302 (196–451)	175 (66–385)

Data are presented as median (interquartile range) for continuous variables and n (%) for categorical variables.* Statistical comparisons between groups are presented in Tables 2–4. CK, creatine kinase; CRP, C-reactive protein; LDH, lactate dehydrogenase; Alb, albumin; KL-6, Krebs von den Lungen-6; PSL, prednisolone; IS, immunosuppressants; IVIG, intravenous immunoglobulin; CNI, calcineurin inhibitor; AZA, azathioprine; MTX, methotrexate; MMF, mycophenolate mofetil; RTX, rituximab; JAK-i, Janus kinase inhibitor; PM, polymyositis; DM, dermatomyositis; CADM, clinically amyopathic dermatomyositis; ASS, anti-synthetase syndrome; ARS, aminoacyl-tRNA synthetase; MDA5, melanoma differentiation-associated gene 5.

At the time of relapse, the median daily prednisolone dose was 7 mg/day (IQR: 4–10 mg/day). Initial treatment strategies included prednisolone monotherapy in 23% of patients, triple therapy (high-dose glucocorticoids, calcineurin inhibitors and intravenous cyclophosphamide) in 14% of patients and combination therapy with prednisolone plus other immunosuppressants in 63% of patients. The most commonly used immunosuppressants were calcineurin inhibitors (71%), followed by intravenous cyclophosphamide (15%) and azathioprine (4%). Intravenous immunoglobulin was administered to 7% of the patients.

### Univariate analysis of risk factors for relapse

Univariate Cox regression analysis identified several factors associated with relapse risk (Table 2). A higher initial prednisolone dose was significantly associated with an increased relapse risk (hazard ratio [HR]: 1.02 per 1 mg/day increase, 95% confidence interval [CI]: 1.01–1.04, *P* = 0.001). Conversely, female sex (HR: 0.52, 95% CI: 0.30–0.92, *P* = 0.024) and higher pre-treatment serum albumin levels (HR: 0.53 per 1 g/dl increase, 95% CI: 0.32–0.87, *P* = 0.012) were associated with a lower relapse risk. Age was not significantly associated with relapse (HR, 1.01; 95% CI: 0.99–1.03, *P* = 0.35).

**Table 2:** Univariate Cox regression analysis of risk factors for relapse in idiopathic inflammatory myopathies.

	All relapse			ILD relapse			Muscle/skin relapse		
Variable	HR	95% CI	*P*-value	HR	95% CI	*P*-value	HR	95% CI	*P*-value
Age (per 1 year)	1.01	0.99–1.03	0.35	1.01	0.99–1.03	0.53	1.01	0.99–1.03	0.48
Female	0.52	0.30–0.92	**0.024**	0.56	0.34–0.93	**0.026**	0.49	0.29–0.83	**0.009**
ILD	1.02	0.52–1.98	0.95	0.79	0.45–1.39	0.42	1.56	0.80–3.08	0.18
Muscle weakness	1.28	0.76–2.16	0.35	1.83	1.07–3.14	**0.027**	1.12	0.72–1.75	0.62
Gottron’s sign	0.98	0.55–1.73	0.94	0.91	0.55–1.50	0.71	0.85	0.53–1.36	0.49
Heliotrope rash	0.89	0.44–1.77	0.73	0.89	0.47–1.66	0.70	0.96	0.54–1.73	0.90
V neck sign	0.92	0.50–1.70	0.80	1.10	0.65–1.89	0.71	0.85	0.49–1.46	0.55
CK (IU/l)	1.000	0.99–1.00	0.21	1.000	0.99–1.00	**0.035**	1.000	0.99–1.00	0.24
CRP (per 1 mg/dl)	1.03	0.87–1.18	0.67	1.03	0.88–1.16	0.72	1.03	0.90–1.16	0.60
Ferritin (per 1 ng/ml)	1.000	0.999–1.000	0.057	1.000	0.999–1.000	0.065	1.000	0.999–1.000	0.14
LDH (per 1 IU/l)	1.001	0.999–1.000	0.25	1.002	1.001–1.003	**0.015**	1.001	0.999–1.002	0.16
Albumin (per 1 g/dl)	0.53	0.32–0.87	**0.012**	0.54	0.33–0.89	**0.016**	0.52	0.33–0.82	**0.005**
KL-6 (per 1 U/ml)	1.000	0.999–1.000	0.28	1.000	0.999–1.000	0.72	1.000	0.999–1.000	0.29
Pre-treatment disease duration	1.003	0.996–1.011	0.35	1.001	0.993–1.007	0.79	1.000	0.993–1.007	0.92
Initial PSL dose (per 1 mg/day)	1.02	1.01–1.04	**0.001**	1.02	0.99–1.03	0.073	1.02	1.01–1.04	**0.002**

Hazard ratios (HR) and 95% confidence intervals (CI) were calculated using univariate Cox proportional hazards regression models. Analysis was performed for all relapses, interstitial lung disease (ILD) relapse and muscle/skin relapse separately. Significant *P*-values (<0.05) are shown in bold. CK, creatine kinase; CRP, C-reactive protein; LDH, lactate dehydrogenase; PSL, prednisolone.

### Multivariate analysis of risk factors

Multivariate Cox regression analysis was performed to identify the independent risk factors for relapse (Table 3). For all relapses, the model included age, sex, initial prednisolone dose and pretreatment albumin level. In this model, only pretreatment albumin levels remained a significant independent predictor of relapse (HR: 0.60 per 1 g/dl increase, 95% CI: 0.40–0.92, *P* = 0.019). Female sex showed a trend toward a lower relapse risk but did not reach statistical significance (HR: 0.63, 95% CI: 0.16–2.52, *P* = 0.071). The initial prednisolone dose (HR: 1.01, 95% CI: 1.00–1.03, *P* = 0.13) and age (HR: 1.01, 95% CI: 0.98–1.02, *P* = 0.94) were not significantly associated with relapse after adjusting for other variables.

**Table 3:** Multivariate Cox regression analysis of risk factors for relapse.

Variable	HR	95% CI	*P*-value
**All relapse**			
Age (per 1 year)	0.999	0.973–1.017	0.96
Female	0.67	0.34–1.30	0.24
Initial PSL dose (per 1 mg/day)	1.028	1.004–1.053	**0.019**
Albumin (per 1 g/dl)	0.51	0.29–0.83	**0.019**
Pre-treatment disease duration (per 1 day)	1.008	0.999–1.017	0.060
**ILD relapse**			
Age (per 1 year)	0.997	0.97–1.02	0.83
Female	0.52	0.31–0.91	**0.022**
Albumin (per 1 g/dl)	0.49	0.30–0.81	**0.0049**
Pre-treatment disease duration (per 1 day)	1.001	0.993–1.008	0.68
**Muscle/skin relapse**			
**Age (per 1 year)**	1.000	0.978–1.025	0.98
Female	0.55	0.32–0.94	**0.030**
Albumin (per 1 g/dl)	0.49	0.30–0.79	**0.0038**
Pre-treatment disease duration (per 1 day)	1.002	0.993–1.009	0.62

Multivariate Cox regression analysis adjusting for clinically important variables and those significant in univariate analysis. All relapse model: age, sex, initial PSL dose, albumin. ILD relapse and muscle/skin relapse models: age, sex, albumin. Values shown are hazard ratios with 95% confidence intervals. *P*-values <0.05 are shown in bold. PSL, prednisolone.

### Serum cytokine profiles and baseline clinical characteristics associated with pretreatment albumin

Among 71 patients with available cytokine data, 16 cytokines showed significant differences between the low and high albumin groups. After applying false discovery rate (FDR) correction using the Benjamini–Hochberg method, 14 cytokines remained statistically significant (q < 0.05) (Supplementary Table S1, available at *Rheumatology Advances in Practice* Online). Patients with low albumin demonstrated marked elevation of IL-6 (7.5-fold, *P* < 0.0001), IP-10/CXCL10 (5.3-fold, *P* < 0.0001), MCP-1/CCL2 (2.5-fold, *P* < 0.0001) and TNF-α (1.9-fold, *P* = 0.0026). Additional pro-inflammatory mediators, including IL-8, IL-1RA and multiple chemokines, were significantly elevated. The vascular adhesion molecules VCAM-1 (*P* < 0.0001) and ICAM-1 (*P* = 0.017) were also increased, indicating endothelial activation.

When patients were stratified according to the median pretreatment serum albumin level (3.65 g/dl), those with lower albumin levels (<3.65 g/dl) were significantly older and exhibited higher levels of inflammatory markers, including creatine kinase, lactate dehydrogenase, C-reactive protein and ferritin, compared with patients with albumin ≥3.65 g/dl (Supplementary Table S2, available at *Rheumatology Advances in Practice* Online). A trend toward higher initial prednisolone doses was also observed in the low-albumin group. These findings indicate that lower pretreatment albumin levels are associated with greater systemic inflammatory burden at baseline.

### Risk factors for interstitial lung disease relapse

In patients who experienced ILD relapse, univariate analysis revealed distinct risk factors (Table 2). The presence of muscle weakness at baseline was associated with an increased risk of ILD relapse (HR: 1.83, 95% CI: 1.07–3.14, *P* = 0.027). Elevated lactate dehydrogenase (LDH) levels were marginally associated with ILD relapse (HR: 1.002 per 1 IU/l increase, 95% CI: 1.001–1.003, *P* = 0.015). Similar to the overall relapse analysis, lower pre-treatment albumin levels were associated with an increased risk of ILD relapse (HR: 0.54 per 1 g/dl increase, 95% CI: 0.33–0.89, *P* = 0.016).

In the multivariate Cox regression analysis, including age, sex and pretreatment albumin level, both albumin (HR: 0.51 per 1 g/dl increase, 95% CI: 0.30–0.88, *P* = 0.015) and female sex (HR: 0.70, 95% CI: 0.52–0.95, *P* = 0.022) remained significant independent predictors of ILD relapse (Table 3). Age was not significantly associated with ILD relapse (HR, 0.995; 95% CI: 0.97–1.02, *P* = 0.72) (Table 3). These findings indicate that lower pre-treatment albumin levels and male sex are independent risk factors for ILD relapse.

### Risk factors for muscle and skin relapse

For relapses involving muscle and skin manifestations, the univariate analysis revealed risk factors similar to those identified in the overall relapse analysis (Table 2). Higher initial prednisolone doses were significantly associated with an increased relapse risk (HR: 1.02 per 1 mg/day increase, 95% CI: 1.01–1.04, *P* = 0.002). Female sex (HR: 0.49, 95% CI: 0.29–0.83, *P* = 0.009) and higher pre-treatment albumin levels (HR: 0.52 per 1 g/dl increase, 95% CI: 0.33–0.82, *P* = 0.005) were protective factors against muscle and skin relapse.

In the multivariate Cox regression analysis, including age, sex and pretreatment albumin level, both albumin (HR: 0.54 per 1 g/dl increase, 95% CI: 0.33–0.87, *P* = 0.012) and female sex (HR: 0.48, 95% CI: 0.27–0.83, *P* = 0.009) remained significant independent predictors of muscle and skin relapse (Table 3). Age was not significantly associated with muscle or skin relapse (HR: 0.999, 95% CI: 0.98–1.02, *P* = 0.94) (Table 3). These results demonstrate that lower pre-treatment albumin levels and male sex are independent risk factors for muscle and skin relapse, with a stronger protective effect in females than in males compared with ILD relapse.

### Analysis in anti-ARS antibody-positive patients

Univariate Cox regression analysis in the anti-ARS antibody-positive subgroup revealed several significant associations (Table 4). For all relapses, the V-neck sign was significantly associated with an increased relapse risk (HR: 4.10, 95% CI: 1.27–13.3, *P* = 0.008). This association was particularly pronounced in ILD relapse, where the V-neck sign demonstrated a very strong effect (HR: 6.05, 95% CI: 2.34–15.6, *P* = 0.006). Additionally, lower pretreatment albumin levels were significantly associated with ILD relapse in this subgroup (HR, 0.49 per 1 g/dl increase; 95% CI: 0.25–0.91, *P* = 0.012). For muscle and skin relapse, the V-neck sign showed a trend but did not reach statistical significance (HR: 2.54, 95% CI: 0.90–7.17, *P* = 0.079). Age and sex were not significantly associated with relapse in any of the analyses within this subgroup.

**Table 4:** Antibody-specific analysis: univariate Cox regression for relapse risk.

Antibody/relapse type	Variable	HR	95% CI	*P*-value
**Anti-ARS antibody positive**				
**All relapse**	Age (per 1 year)	1.01	0.98–1.05	0.56
	Female	0.61	0.28–1.32	0.21
	Albumin (per 1 g/dl)	0.66	0.32–1.39	0.25
	V-neck sign	4.10	1.27–13.3	**0.008**
**ILD relapse**	Age (per 1 year)	1.006	0.97–1.04	0.76
	Female	0.63	0.31–1.27	0.20
	Albumin (per 1 g/dl)	0.49	0.25–0.91	**0.012**
	V-neck sign	6.05	2.34–15.6	**0.006**
**Muscle/skin relapse**	Age (per 1 year)	1.01	0.98–1.04	0.42
	Female	0.95	0.47–1.90	0.88
	Albumin (per 1 g/dl)	0.66	0.34–1.25	0.21
	V-neck sign	2.54	0.90–7.17	0.079
**Anti-MDA5 antibody positive**				
**ILD relapse**	Gottron’s sign	0.05	0.004–0.54	**0.014**
	Mechanic’s hand	0.31	0.09–1.05	0.060

Univariate Cox proportional hazards regression analysis stratified by myositis-specific autoantibody subgroups. For anti-ARS antibody-positive patients (*n* = 53, 24 relapse events), analyses were performed for all relapses, ILD relapses and muscle/skin relapses, examining age, sex, pretreatment albumin level and V-neck sign. For anti-MDA5 antibody-positive patients (*n* = 21, six relapse events), the analysis focused on ILD relapse (*n* = 5 events) and examined Gottron’s sign and mechanic’s hand. Hazard ratios (HR) with 95% confidence intervals (CI) are shown. Significant *P*-values (<0.05) are bolded. These univariate findings correspond to the multivariate models presented in Table 5. ARS, aminoacyl-tRNA synthetase; MDA5, melanoma differentiation-associated gene 5; ILD, interstitial lung disease.

To identify independent predictors within the anti-ARS antibody-positive subgroup, multivariate Cox regression analyses were performed for all relapses, ILD relapses and muscle/skin relapses (Table 5). For all relapses in anti-ARS-positive patients, both pretreatment albumin level (HR: 0.56 per 1 g/dl increase, 95% CI: 0.31–0.98, *P* = 0.043) and V-neck sign (HR: 1.73, 95% CI: 1.06–2.76, *P* = 0.029) remained significant independent predictors after adjusting for age, sex, albumin and V-neck sign. In the ILD relapse analysis for anti-ARS-positive patients, the V-neck sign emerged as a particularly strong independent predictor (HR: 5.07, 95% CI: 1.64–15.75, *P* = 0.006), while albumin showed only a trend (HR: 0.59, 95% CI: 0.29–1.14, *P* = 0.12). This finding indicates that the V-neck sign is specifically and powerfully associated with ILD progression in this subgroup. In contrast, for muscle and skin relapse among anti-ARS-positive patients, no variables reached statistical significance in the multivariate model.

**Table 5:** Antibody-specific multivariate Cox regression analysis of risk factors for relapse.

Antibody/relapse type	Variable	HR	95% CI	*P*-value
**Anti-ARS antibody positive**				
**All relapse**	Age (per 1 year)	0.997	0.970–1.026	0.83
	Female	1.03	0.73–1.46	0.88
	Albumin (per 1 g/dl)	0.56	0.31–0.98	**0.043**
	V-neck sign	1.73	1.06–2.76	**0.029**
**ILD relapse**	Age (per 1 year)	0.99	0.96–1.03	0.54
	Female	0.92	0.61–1.41	0.71
	Albumin (per 1 g/dl)	0.59	0.30–1.14	0.12
	V-neck sign	5.07	1.64–15.8	**0.006**
**Muscle/skin relapse**	Age (per 1 year)	1.01	0.98–1.04	0.69
	Female	0.92	0.59–1.46	0.72
	Albumin (per 1 g/dl)	0.77	0.37–1.58	0.48
	V-neck sign	1.36	0.80–2.58	0.20
**Anti-MDA5 antibody positive**				
**ILD relapse**	Gottron’s sign	0.07	0.006–0.88	**0.039**
	Mechanic’s hand	0.61	0.31–1.17	0.14

Multivariate Cox proportional hazards regression analysis was stratified according to the autoantibody subgroups. For anti-ARS antibody-positive patients (*n* = 53, 24 relapse events), all models included age, sex, pretreatment albumin level and V-neck sign. Analyses were performed separately for all relapses, ILD relapses and muscle/skin relapses. For anti-MDA5 antibody-positive patients (*n* = 21, six relapse events), the analysis focused on ILD relapse (*n* = 5 events) and included Gottron’s sign and mechanic’s hand. Owing to the limited number of events, this should be considered an exploratory analysis that requires validation in larger cohorts. The variables were selected based on clinical importance and univariate significance (Table 4). Hazard ratios (HR) with 95% confidence intervals (CI) are shown. Significant *P*-values (<0.05) are bolded. ARS, aminoacyl-tRNA synthetase; MDA5, melanoma differentiation-associated gene 5; ILD, interstitial lung disease.

### Analysis in anti-MDA5 antibody-positive patients

In the anti-MDA5 antibody-positive subgroup, given that the majority of relapses were ILD-related (5 of 6 cases, 83%), univariate and multivariate analyses focused on ILD relapse (Tables 4 and 5). In the univariate analysis, Gottron’s sign demonstrated a strong protective effect against ILD relapse (HR: 0.05, 95% CI: 0.004–0.54, *P* = 0.014), while mechanic’s hand showed a trend toward protection that did not reach statistical significance (HR: 0.31, 95% CI: 0.09–1.05, *P* = 0.060). Age, sex and pretreatment albumin levels were not significantly associated with relapse in this subgroup, although the limited number of events restricted the statistical power of these analyses.

Due to the limited number of events (*n* = 5), we performed an exploratory multivariate analysis specifically for ILD relapse, including only Gottron’s sign and mechanic’s hand. In this exploratory analysis, Gottron’s sign remained a strong independent protective factor against ILD relapse (HR: 0.07, 95% CI: 0.01–0.88, *P* = 0.039), whereas mechanic’s hand showed a trend but did not reach statistical significance (HR: 0.61, 95% CI: 0.31–1.17, *P* = 0.14). However, given the small sample size and wide confidence intervals, these findings should be interpreted with caution and require validation in larger cohorts in future studies.

## Discussion

This retrospective study identified several significant risk factors for relapse in patients with IIM through comprehensive univariate and multivariate analyses of clinical characteristics, laboratory parameters and treatment patterns. Our findings provide important insights into the prediction and management of IIM relapses, with particular emphasis on independent predictors that remained significant after adjusting for potential confounders.

One of the most notable findings of our study was the consistent association between pretreatment serum albumin levels and relapse risk across different manifestations. In the multivariate analysis, albumin remained the only independent predictor of overall relapse and maintained significance for both ILD and muscle/skin relapse after adjustment for age, sex and other confounders. This robust finding across multiple analytical models suggests that serum albumin level serves as a powerful biomarker for disease stability in IIMs.

Our cytokine analysis revealed that patients with low albumin levels had a profound elevation in inflammatory mediators, particularly IL-6, IP-10 and MCP-1. The marked elevation of IL-6, a central mediator of acute phase responses, suggests the ongoing activation of inflammatory pathways through the IL-6/JAK/STAT3 signaling cascade. This persistent inflammatory state may predispose patients to relapse, even during apparent clinical remission, as subclinical inflammation creates a biological milieu conducive to disease reactivation. The concurrent elevation of multiple chemokines (MCP-1, MCP-3, IL-8) indicates an enhanced immune cell recruitment capacity, whereas elevated adhesion molecules (VCAM-1, ICAM-1) suggest vascular endothelial activation, facilitating leucocyte extravasation.

Low albumin levels are generally associated with inflammation and poor nutritional status [14], and in the context of IIMs, they may reflect ongoing subclinical inflammation, even in apparently stable patients. The relationship between albumin and disease activity could be mediated through multiple mechanisms, including its role in maintaining oncotic pressure, binding and transporting various molecules and its anti-inflammatory property. This finding is particularly valuable for clinical practice, as serum albumin is routinely measured and can be readily incorporated into risk assessment protocols.

Our findings complement those of the large JAMI cohort study [15], which established CRP and KL-6 levels as independent predictors of mortality in patients with PM/DM-ILD. Notably, the JAMI study focused on survival outcomes, whereas our study specifically examined relapse as the primary endpoint. This difference in outcomes explains why CRP, a marker of acute inflammation associated with short-term mortality risk, did not emerge as a significant predictor of relapse in the present cohort. In contrast, pretreatment albumin, which was not examined in the JAMI study, emerged as an independent predictor of relapse in our multivariate analysis. Our cytokine profiling suggests that while acute inflammatory markers, such as CRP, predict immediate severe outcomes, albumin serves as an integrated biomarker of chronic systemic inflammation that predisposes patients to disease relapse over time. The biological plausibility is supported by our finding that patients with low albumin levels have elevated IL-6 levels, which is known to suppress albumin synthesis while driving chronic inflammation.

Although ILD relapse-free survival did not differ significantly between anti-ARS and anti-MDA5 antibodies, our findings suggest that relapse risk is better stratified by antibody-specific clinical phenotypes rather than antibody status alone. Multivariate analysis within antibody-defined subgroups demonstrated fundamentally different risk-factor profiles. In anti-ARS-positive patients, the V-neck sign emerged as a particularly powerful independent predictor of ILD relapse, representing the strongest risk factor identified in our study. This association remained robust even after adjusting for age, sex and albumin level. Conversely, in anti-MDA5-positive patients, Gottron’s sign demonstrated a strong independent protective effect against ILD relapse in exploratory multivariate analysis, although this finding should be interpreted cautiously, given the limited sample size.

These contrasting associations of cutaneous manifestations—with the V-neck sign predicting worse outcomes in anti-ARS patients and Gottron’s sign predicting better outcomes in anti-MDA5 patients—strongly suggest fundamentally different pathophysiological mechanisms underlying disease relapse in these antibody-defined subtypes. These findings extend beyond previous reports of antibody-specific phenotypes [16, 17] to demonstrate that risk stratification strategies should be tailored according to autoantibody status, with different clinical signs carrying opposite prognostic implications depending on the underlying autoantibody profile.

Initial treatment strategies did not differ substantially between patients with and without relapse. Although patients who later experienced relapse more frequently received intravenous immunoglobulin and higher initial prednisolone doses, these treatments likely reflected greater disease severity at baseline. In multivariable analyses, treatment-related variables were not independently associated with relapse, whereas pretreatment albumin level remained a robust predictor. This finding aligns with previous research demonstrating that initial disease severity influences IIM outcomes [18, 19]. Treatment intensity may not independently predict relapse when the severity is adequately captured by variables such as albumin levels. In addition, female sex independently predicted a reduced ILD relapse risk in our cohort. This protective effect likely reflects the interplay between sex hormones and autoimmune regulation, similar to the patterns observed in other autoimmune diseases [20]. The immunomodulatory effects of estrogen on T cell differentiation and cytokine production may contribute to this protection, potentially informing personalized treatment strategies.

Our study had several limitations that should be considered. The retrospective, single-center design may limit the generalizability of our findings and introduce a selection bias. The relatively small sample size, particularly in antibody-specific subgroups, limited our statistical power, as evidenced by the wide confidence intervals in some multivariate analyses. The anti-MDA5 subgroup analysis was particularly constrained by the small number of events (*n* = 5 ILD relapses) and thus should be considered exploratory and require validation in larger cohorts. Furthermore, the definition of relapse, although clinically relevant, relied on physician judgment and may have introduced some subjectivity. The lack of standardized quantitative criteria for relapse across all domains (biochemical, clinical and imaging) is another limitation. Finally, our focus on patients who achieved remission after the initial treatment introduced selection bias. By excluding 8 patients who died before achieving remission, we specifically examined relapse prediction in survivors, which represents a clinical scenario distinct from mortality prediction. This survival bias indicates that our findings are most applicable to patients who successfully achieve initial disease control.

In conclusion, pretreatment serum albumin levels independently predict relapse in patients with IIMs, with mechanistic support from cytokine profiling demonstrating that low albumin levels reflect active systemic inflammation. The divergent prognostic value of cutaneous signs across antibody subgroups highlights the pathogenic heterogeneity of IIMs and supports antibody-based personalized approaches for monitoring and treatment.

## Supplementary Material

rkag021_Supplementary_Data

## Data Availability

The datasets used and/or analyzed during the current study are available from the corresponding author upon reasonable request, subject to appropriate ethical approval and data-sharing agreements.
