# Supplementary material for: Pre-treatment serum albumin predicts relapse in idiopathic inflammatory myopathies: a retrospective cohort study with cytokine profiling
Source: Rheumatol Adv Pract. 2026 Feb 5;10(2):rkag021. doi: 10.1093/rap/rkag021 (PMC13006065; doi:10.1093/rap/rkag021)
Supplement: rkag021_Supplementary_Data [file rkag021_supplementary_data.zip › Supplementary Table S1.docx]

**Supplementary Table S1. Serum cytokine levels stratified by pretreatment albumin status**

| **Cytokine** | **P-value** | **q-value  (FDR)** | **Low Albumin  (<3.65 g/dL) Median** | **25th  Percentile** | **75th  Percentile** | **High Albumin (≥3.65 g/dL) Median** | **25th  Percentile** | **75th  Percentile** | **Fold Change** |
| --- | --- | --- | --- | --- | --- | --- | --- | --- | --- |
| **IL-6** | 0.0001 | 0.0006 | 15.16 | 4.92 | 23.24 | 2.02 | 1.25 | 5.59 | 7.5 |
| **IP-10 (CXCL10)** | 0.0001 | 0.0006 | 10298.82 | 4282.05 | 19012.92 | 1936.2 | 870.68 | 5652.88 | 5.3 |
| **MCP-1 (CCL2)** | 0.0001 | 0.0006 | 1996.84 | 1461.33 | 2984.1 | 802.52 | 636.07 | 1303.44 | 2.5 |
| **MCP-3 (CCL7)** | 0.0001 | 0.0006 | 53.27 | 29.68 | 106.23 | 10.87 | 5.24 | 47.51 | 4.9 |
| **VCAM-1** | 0.0001 | 0.0006 | 2362400 | 1612450 | 3715550 | 1410050 | 1192750 | 1805000 | 1.7 |
| **IL-1RA** | 0.0001 | 0.0006 | 36.12 | 14.06 | 85.18 | 8.1 | 3.98 | 13.27 | 4.5 |
| **IL-8 (CXCL8)** | 0.0003 | 0.0015 | 21.05 | 12.81 | 30.42 | 10.05 | 6.2 | 17.65 | 2.1 |
| **TNF-α** | 0.0026 | 0.0114 | 47.87 | 24.68 | 94.29 | 25.49 | 14.46 | 48.46 | 1.9 |
| **FLT-3L** | 0.0136 | 0.0490 | 75.77 | 44.63 | 112.67 | 45.64 | 29.56 | 67.49 | 1.7 |
| **IL-15** | 0.0141 | 0.0490 | 19.63 | 13.03 | 30.49 | 12.16 | 7.39 | 20.43 | 1.6 |
| **ICAM-1** | 0.0170 | 0.0490 | 1315250 | 1012872 | 1787500 | 1055550 | 930957 | 1292500 | 1.2 |
| **IL-12p40** | 0.0175 | 0.0490 | 151.52 | 75.98 | 301.06 | 83.92 | 46.85 | 152.08 | 1.8 |
| **IL-18** | 0.0184 | 0.0490 | 43.18 | 22.3 | 102.59 | 25.19 | 11.92 | 42.41 | 1.7 |
| **IL-7** | 0.0196 | 0.0490 | 14.44 | 5.83 | 23.61 | 7.82 | 5.44 | 11.68 | 1.8 |
| GROα (CXCL1) | 0.0257 | 0.0600 | 46.54 | 30.19 | 76.41 | 30.58 | 18.1 | 45.73 | 1.5 |
| IL-27 | 0.0320 | 0.0700 | 2183.99 | 1434.05 | 3184.09 | 1533.31 | 919.16 | 2591.44 | 1.4 |
| Eotaxin (CCL11) | 0.0528 | 0.1077 | 163.11 | 123.34 | 202.76 | 134.06 | 87.27 | 174.6 | 1.2 |
| FGF-2 | 0.0554 | 0.1077 | 41.31 | 20.62 | 98.44 | 30.14 | 18.9 | 38.59 | 1.4 |
| MDC (CCL22) | 0.0634 | 0.1168 | 764.25 | 509 | 1005.28 | 875.77 | 671 | 1098.42 | 0.9 |
| IL-17A | 0.1197 | 0.2087 | 4.74 | 1.05 | 18.41 | 2.45 | 0 | 7.53 | 1.9 |
| TNF-β | 0.1252 | 0.2087 | 9.69 | 6.06 | 22.16 | 7.65 | 4.06 | 12.49 | 1.3 |
| IFN-γ | 0.1433 | 0.2280 | 4.12 | 0 | 14.31 | 1.94 | 0 | 7.28 | 2.1 |
| IL-1α | 0.1594 | 0.2326 | 7.24 | 4.24 | 41.29 | 6.23 | 1.9 | 10.88 | 1.2 |
| IL-5 | 0.1595 | 0.2326 | 9.08 | 3.62 | 14.01 | 5.56 | 3.89 | 9.42 | 1.6 |
| Fractalkine (CX3CL1) | 0.1907 | 0.2670 | 140.11 | 99.49 | 275.96 | 113.72 | 79.69 | 211.65 | 1.2 |
| IFN-α2 | 0.2203 | 0.2966 | 12.43 | 0 | 130.24 | 18.62 | 0 | 37.23 | 0.7 |
| VEGF-A | 0.2785 | 0.3610 | 382.43 | 217.37 | 574.35 | 340.6 | 169.06 | 486.22 | 1.1 |
| IL-13 | 0.3860 | 0.4825 | 51.44 | 17.17 | 198.36 | 47.3 | 19.85 | 82.98 | 1.1 |
| TGF-α | 0.4467 | 0.5391 | 4.37 | 1.8 | 15.54 | 3.98 | 1.96 | 6.69 | 1.1 |
| sCD40L | 0.5037 | 0.5877 | 5817.12 | 2697.95 | 9385.35 | 6886.02 | 4982.74 | 9539.64 | 0.8 |
| MIP-1α (CCL3) | 0.5411 | 0.6002 | 27.79 | 16.37 | 47.44 | 25.68 | 13.55 | 34.93 | 1.1 |
| PDGF-AA | 0.5488 | 0.6002 | 4870.41 | 3283.68 | 6254.97 | 4924.86 | 4238.39 | 5976.61 | 1 |
| MIP-1β (CCL4) | 0.7122 | 0.7554 | 38.58 | 21.94 | 53.44 | 37.16 | 28.14 | 52.52 | 1 |
| IL-1β | 0.8670 | 0.8925 | 10.1 | 2.49 | 52.17 | 11.53 | 4.4 | 21.5 | 0.9 |
| EGF | 0.9357 | 0.9357 | 105.25 | 64.29 | 174.63 | 124.89 | 65.83 | 160.94 | 0.8 |

Cytokine concentrations were measured in 71 patients using a multiplex immunoassay. Patients were stratified according to the median pretreatment albumin level (3.65 g/dL). Data are presented as median values with interquartile ranges (25th–75th percentile). P-values were calculated using the Mann–Whitney U test. Q-values represent false discovery rate (FDR)–adjusted P-values using the Benjamini–Hochberg procedure to account for multiple comparisons. Fold change represents the ratio of median cytokine levels in the low-albumin group (<3.65 g/dL) relative to the high-albumin group (≥3.65 g/dL). L, interleukin; IP-10 (CXCL10), interferon-γ–induced protein 10; MCP-1 (CCL2), monocyte chemoattractant protein-1; MCP-3 (CCL7), monocyte chemoattractant protein-3; VCAM-1, vascular cell adhesion molecule-1; IL-1RA, interleukin-1 receptor antagonist; IL-8 (CXCL8), interleukin-8; TNF-α, tumor necrosis factor-α; FLT-3L, Fms-like tyrosine kinase 3 ligand; ICAM-1, intercellular adhesion molecule-1; IL-12p40, interleukin-12 subunit p40; GROα (CXCL1), growth-regulated oncogene-α; IL-27, interleukin-27; MDC (CCL22), macrophage-derived chemokine; TNF-β, tumor necrosis factor-β; IFN-γ, interferon-γ; Fractalkine (CX3CL1); IFN-α2, interferon-α2; VEGF-A, vascular endothelial growth factor-A; TGF-α, transforming growth factor-α; sCD40L, soluble CD40 ligand; MIP-1α (CCL3), macrophage inflammatory protein-1α; PDGF-AA, platelet-derived growth factor-AA; MIP-1β (CCL4), macrophage inflammatory protein-1β; EGF, epidermal growth factor.
