# Supplementary material for: Pre-treatment serum albumin predicts relapse in idiopathic inflammatory myopathies: a retrospective cohort study with cytokine profiling
Source: Rheumatol Adv Pract. 2026 Feb 5;10(2):rkag021. doi: 10.1093/rap/rkag021 (PMC13006065; doi:10.1093/rap/rkag021)
Supplement: rkag021_Supplementary_Data [file rkag021_supplementary_data.zip › Supplementary Table S2.docx]

**Supplementary Table S2. Baseline clinical characteristics stratified by pretreatment serum albumin level using the median cutoff (3.65 g/dL)**

| **Variable** | **Albumin <3.65 g/dL (n = 44)** | **Albumin ≥3.65 g/dL (n = 44)** | **P value** |
| --- | --- | --- | --- |
| Age, years | 65 (54.3-71.8) | 58 (50.3-63) | **0.0032** |
| Female sex, n (%) | 27 (61) | 27 (61) | 1 |
| Interstitial lung disease at diagnosis, n (%) | 39 (88) | 33 (75) | 0.17 |
| Creatine kinase (U/L) | 523 (157-1612) | 217 (81-582) | **0.012** |
| Lactate dehydrogenase (U/L) | 372 (322-457) | 257 (208-345) | **<0.0001** |
| C-reactive protein (mg/dL) | 1.3 (0.16-2.3) | 0.2 (0.06-0.5) | **<0.0001** |
| Ferritin (ng/mL) | 432 (211-850) | 163 (78-443) | **0.0044** |
| Initial prednisolone dose (mg/day) | 40 (40-54) | 40 (30-50) | 0.054 |

Data are presented as median (interquartile range) or number (%), as appropriate. Comparisons between groups were performed using the Wilcoxon rank-sum test for continuous variables and the chi-square or Fisher’s exact test for categorical variables, as appropriate. Missing data were excluded from each analysis.
